# Persistent tissue‐specific resident microbiota in oysters across a broad geographical range

**DOI:** 10.1111/1758-2229.70026

**Published:** 2024-10-24

**Authors:** Andrea Unzueta‐Martínez, Jennifer Bowen

**Affiliations:** ^1^ Department of Marine and Environmental Science Northeastern University Nahant Massachusetts USA; ^2^ Present address: Department of Organismic and Evolutionary Biology Harvard University Cambridge Massachusetts USA

## Abstract

Marine animals often harbour complex microbial communities that influence their physiology. However, strong evidence for resident microbiomes in marine bivalves is lacking, despite their contribution to estuarine habitats and coastal economies. We investigated whether marine bivalves harbour stable, resident microorganisms in specific tissues or if their microbiomes primarily consist of transient members reflecting the environmental microbial pool. Conducting a latitudinal study of wild eastern oysters (*Crassostrea virginica*) along the East Coast of the United States, we aimed to identify resident microorganisms that persist across a wide geographical range. Our results revealed that microbial communities in seawater and sediment samples followed latitudinal diversity patterns driven by geographic location. In contrast, oyster‐associated microbiomes were distinct from their surrounding environments and exhibited tissue‐specific compositions. Notably, oyster microbiomes showed greater similarity within the same tissue type across different geographic locations than among different tissue types within the same location. This indicates the presence of tissue‐specific resident microbes that persist across large geographical ranges. We identified a persistent set of resident microbiome members for each tissue type, with key microbial members consistent across all locations. These findings underscore the oyster host's role in selecting its microbiome and highlight the importance of tissue‐specific microbial communities in understanding bivalve‐associated microbiomes.

## Graphical abstract

Wild eastern oysters along the US East Coast harbour unique, tissue‐specific microbial communities that are distinct from the surrounding seawater and sediment microbiomes. These resident microbes persist across different geographic locations, underscoring the oyster's role in selecting its microbiome. Our findings enhance the understanding of bivalve‐associated microbiomes and their ecological significance.

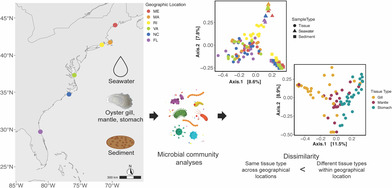

## INTRODUCTION

Many animals have associated microbiomes that are made up of a variety of eukaryotic and prokaryotic organisms, including bacteria, archaea, viruses, fungi, and protozoans that can reside on or within the host. The term ‘microbiome’ (Lederberg & McCray, 2001) is broad and encompasses different kinds of microbial associations with their host. Hammer et al. (2019) proposed to use residency, the extent to which a microbial population is stably associated with a host, to tease apart members of the microbiome that may be relevant to host physiology from those that are inconsequential. Resident microbes are distinct from transient microbes in that they reproduce within a host at a rate higher than their loss due to death or excretion creating stable patterns of abundance and membership (Harris, 1993). Ecologists and microbiologists have long emphasized the importance of distinguishing between resident and transient members of communities (Berg, 1996; Harris, 1993; Snell Taylor et al., 2018). More recently, animal microbiome studies also focused on differentiating resident from transient microbes (Auchtung et al., 2018; David et al., 2014; Hammer et al., 2017; Lee et al., 2016; Unzueta‐Martínez, Welch, & Bowen, 2022). It is essential to apply these concepts to non‐model organisms to better understand broad patterns in host‐associated microbial community ecology.

Historically, microbes associated with marine bivalves were of interest to biologists, for example, the prokaryotes associated with oysters have been studied for over a century (Round, 1914). Bivalves, particularly oysters, attract significant attention because they provide essential ecosystem services, as well as sustenance and income to coastal communities across the globe. For example, the eastern oyster (*Crassostrea virginica*) fishery generates over $196 million dollars annually in the United States alone (NMFS, 2015). Thus, understanding factors that influence the health of bivalves has important environmental and economic implications. However, most research focuses on bacteria pathogenic to humans (Rippey, 1994), rather than bacteria that may be relevant to the host bivalve itself. Additionally, homogenization of the whole animal was routine, and the use of culture‐dependent methods limited the evaluation of bivalve microbiota (Murcherlano & Brown, 1968). Bivalve‐associated microbial community ecology studies are now possible using molecular techniques (e.g., next‐generation sequencing), which allows for the characterization of tissue‐specific bivalve microbiomes across spatial and temporal scales.

Investigations of marine bivalve microbiomes lead to mixed results. Some studies report highly consistent, species‐specific bacterial communities associated with bivalve hosts, regardless of geography (Roterman et al., 2015; Zurel et al., 2011), a characteristic that may indicate an important association with the host. Other studies report high intraspecific variability in microbial community composition between geographic locations (King et al., 2012) and higher interspecific microbiome similarity at the same site than to conspecifics at different sites (Trabal et al., 2012), patterns that may indicate loose associations with the animal host. These inconsistencies may be due to methodological artefacts such as contamination of low‐biomass samples (Eisenhofer et al., 2019), seasonal differences at the time of sampling, starvation or feeding of the bivalve before sampling (Harris, 1993), sequencing of extracellular DNA, or the sampling of hatchery‐raised animals (Ishaq et al., 2023). Additionally, many bivalve microbiome surveys do not distinguish between loosely‐associated (‘transient’) from the stably associated (‘resident’) microbes. While specific microbes have been known to have intimate symbiotic associations (reviewed in Hughes & Girguis, 2023) and cause disease in bivalves (Andrews, 1979), the potential importance of the microbial communities residing on bivalve mucosal surfaces as mutualists remains unclear.

To investigate whether marine bivalves have stable, resident microorganisms inhabiting specific tissues or if their microbiomes mostly consist of transient members that reflect the environmental pool of microbes, we conducted a latitudinal study of wild eastern oysters to determine whether there are resident microorganisms that persist across a wide geographical range. We collected seawater, sediment, and oyster tissues (gill, mantle, and stomach), for microbial community analysis from six oyster reefs across the East Coast of the United States. First, we assessed the free‐living microbiomes in the six oyster reefs by determining if (1) sediment and (2) seawater microbial communities were distinct depending on geographic location. Second, we assessed the contribution of the environmental pool of microbes to oyster microbiomes by (3) testing whether oyster microbiomes were distinct from the microbiomes of their surrounding environment. Then, we determined whether (4) tissue type was a stronger predictor of microbial community composition than geographic location, and (5) identified tissue‐specific resident microbial members that persisted across geographic locations.

## EXPERIMENTAL PROCEDURES

### Oyster, seawater, and sediment field collections

**FIGURE 1:**
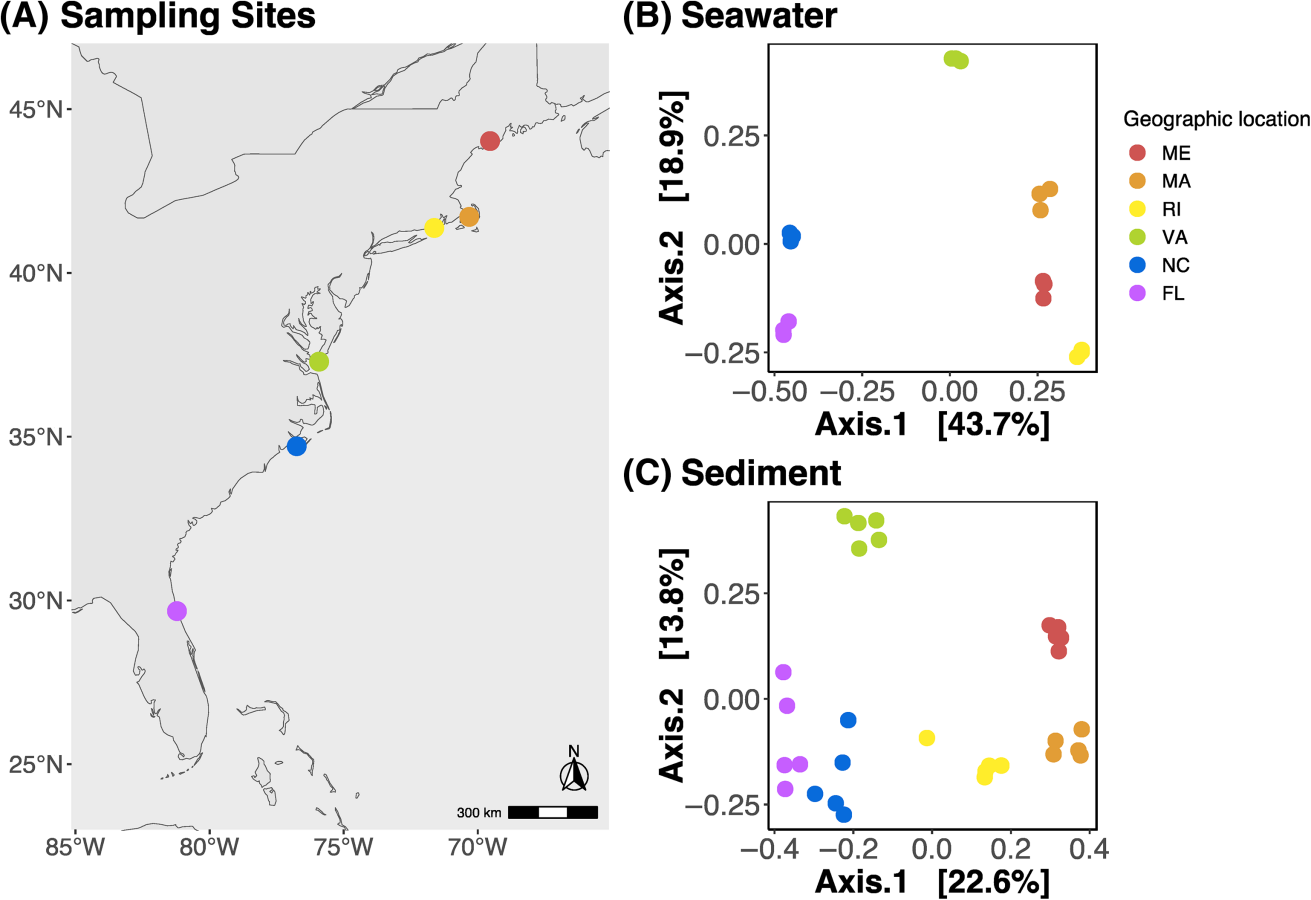
Map of sampling sites and principal component analysis (PCoA) plots of Bray–Curtis dissimilarities of environmental samples. (A) Map of geographic locations of the six oyster reefs sampled on the East Coast of the United States. From North to South: Damariscotta River (44°01′38.1″ N 69°32′35.7″ W) in Maine (ME), Barnstable (41°42′37.6″ N 70°18′18.5″ W) in Massachusetts (MA), Green Hill Pond (41°22′16.1″ N 71°37′13.4″ W) in Rhode Island (RI), Horse Island (37°17′15.5″ N 75°55′02.0″ W) in Virginia (VA), Atlantic Beach (34°42′24.9″ N 76°45′05.7″ W) in North Carolina (NC), and St. Augustine (29°40′17.7″ N 81°12′53.5″ W) in Florida (FL). PCoA of Bray–Curtis Dissimilarity distances of microbial communities in (B) seawater and (C) sediment samples. Sampling geographic locations are indicated across all plots with dots coloured coded in a rainbow gradient based on latitude.

At low tide, we collected adult Eastern Oysters (*C. virginica*) from six different intertidal oyster reefs along the East Coast of the United States (Figure 1A) in the summer of 2018 over the span of 2 weeks. Our sampling sites were at Damariscotta River (44°01′38.1″ N 69°32′35.7″ W) in Maine (ME), Barnstable (41°42′37.6″ N 70°18′18.5″ W) in Massachusetts (MA), Green Hill Pond (41°22′16.1″ N 71°37′13.4″ W) in Rhode Island (RI), Horse Island (37°17′15.5″ N 75°55′02.0″ W) in Virginia (VA), Atlantic Beach (34°42′24.9″ N 76°45′05.7″ W) in North Carolina (NC), and St. Augustine (29°40′17.7″ N 81°12′53.5″ W) in Florida (FL) (Figure 1A). Our goal was to characterize the oyster microbiome as close to as possible to in situ, so at each sampling location we immediately dissected five oysters to collect ~0.25 g of gill (the full set of gill filaments, both inner and outer demibranchs on the ventral side were collected and homogenized), mantle (near the gills opposite to the hinge), and stomach (consisting of stomach and digestive gland tissue, which we refer to as simply ‘stomach’ throughout the rest of the article) from each specimen. We sterilized our dissecting tools using ethanol and rinsed the tissue samples with autoclave‐sterilized water to dislodge any loosely associated microbes. Additionally, we filtered 1 L of site seawater through a 0.22 μm Sterivex filter. Separately, we collected ~0.25 g wet sediments directly adjacent to the oyster reefs by scooping them with a spatula into cryovials. All tissue (*n* = 5 per tissue type), water (*n* = 3), and sediment (*n* = 5) samples were flash frozen in liquid nitrogen and stored at −80°C until DNA extraction.

### DNA preparation and sequencing

We used the DNeasy PowerLyzer PowerSoil kit (Qiagen, Valencia, CA, USA), following the manufacturer's protocol, to extract DNA from tissue, water, and sediment samples. Sterivex filter casings were cracked open with a sterilized PVC pipe cutter, and filters were cut into strips with sterile razor blades to fit them into the bead beading tubes of the DNA extraction kit. Next, we amplified the V4 region of the 16S rRNA gene using the primers 515FY: 5′ TATGGTAATTGTGTGYCAGCMGCCGCGGTAA 3′ (Parada et al., 2016) and 806RB: 3′ AGTCAGTCAGCCGGACTACNVGGGTWTCTAAT 5′ (Apprill et al., 2015) and the 5 PRIME Hot Master Mix (Quanta Bio, Beverly, MA, USA) in triplicate 25 μL polymerase chain reactions (PCR) as previously described (Unzueta‐Martínez, Scanes, et al., 2022). After running the triplicate PCR product and negative controls on a gel to ensure the product matched the target size of ~390 bp and that there was no contamination, we purified and size selected the PCR products using Agencourt AMPure Magnetic Beads (Beckman Coulter, Brea, CA, USA), and resuspended them in 20 μL of nuclease‐free water. We prepared sequencing libraries using Illumina paired‐end adapters with unique Nextera XT v2 indexes as previously described (Unzueta‐Martínez, Scanes, et al., 2022) and purified and once again size selected the PCR products using Agencourt AMPure Magnetic Beads. To quantify our libraries, we used the Quant‐iT PicoGreen dsDNA Assay Kit (Invitrogen, Carlsbad, CA, USA) and pooled samples for sequencing at equimolar concentrations. We also used an Agilent 4200 TapeStation (Agilent Technologies, Santa Clara, CA, USA) and a KAPA library quantification kit (Roche Sequencing Solutions Inc., Pleasanton, CA, USA) to confirm library size and quantify our libraries. We sequenced our library on an Illumina MiSeq with 2 × 250 V2 sequencing chemistry at the Tufts University Core Sequencing Facility.

### Procedural controls

We included DNA extraction negative controls with every extraction batch, and PCR amplification negative controls that were carried through library preparation and sequencing. Additionally, we sequenced three replicates of a mock community (ZymoBIOMICS™ Microbial Community DNA Standard, Zymo Research, USA), with known theoretical relative abundances of 10 species, as a positive control. Figure S1 illustrates that our mock community replicates were highly consistent with their expected composition.

### Sequence analysis

We used DADA2 (v1.7.0) with default settings (Callahan et al., 2016), implemented in R Studio (v4.0.0), to quality‐filter, merge paired‐end reads, remove chimeric sequences, group the sequences into amplicon sequence variants (ASVs), and assign taxonomy against the Silva database (version 132; Quast et al., 2012). We used the Decontam package to identify potential procedural and reagent contaminants based on either the frequency of each ASV as a function of the input DNA concentration or the prevalence of each ASV in true samples compared with the prevalence in negative controls (Davis et al., 2018). We assessed the composition of the mock communities to ensure they agreed with the theoretical composition (Figure S1). We used the Phyloseq package (McMurdie & Holmes, 2013) to filter out ASVs identified as mitochondria, chloroplasts, Eukaryota, and Archaea, which in total accounted for <3% of our dataset. We removed samples that had <1000 reads after quality filtering (*n* = 12 of 138). Rarefaction analyses confirm that the sequencing coverage was sufficient to capture representative bacterial diversity in sediment, seawater, and oyster tissue samples (Figure S2). We used two approaches to account for uneven sequencing depths across samples, we (1) transformed our data to proportions by dividing the reads for each ASV in a sample by the total number of reads in that sample, as previously recommended (Bullard et al., 2010; Dillies et al., 2013; McKnight et al., 2019; McMurdie & Holmes, 2014; Weiss et al., 2017) to conduct β diversity analyses, and (2) normalized reads by converting ASV abundances to *Z*‐scores before running Random Forrest classification models. The rest of our statistical analyses relied on presence/absence data of samples that were sequenced deeply enough to have representative diversity (Figure S2).

### Statistical analyses

To test our first two hypotheses of whether (1) sediment and (2) seawater microbial communities were distinct depending on geographic location, we focused on β diversity and computed Bray–Curtis dissimilarity using the vegdist function in vegan (Oksanen et al., 2020). We ran permutational multivariate analysis of variance (PERMANOVA) with 999 permutations using adonis2 independently for the sediment and seawater samples and tested for homogeneity of group dispersions using the betadisper function in vegan (Oksanen et al., 2020). We visualized the Bray–Curtis dissimilarities of sediment and water samples using principal component analysis (PCoA) plots using the ordinate function in Phyloseq. Additionally, we calculated a linear regression and correlation between the first PCoA axis and latitude using the lm function in base R.

To test whether (3) oyster microbiomes were distinct from the microbiomes of their surrounding environment, we analysed the β diversity of seawater, sediment, and tissue samples. We computed Bray–Curtis dissimilarity using the vegdist function in vegan (Oksanen et al., 2020) and used this dissimilarity matrix to run a single‐factor (with three levels: seawater, sediment, and tissue) PERMANOVA with geographic location as blocks with 999 permutations using adonis2 function in vegan (Oksanen et al., 2020). Permutations were constrained to geographic location using the how function in the permute R package (Simpson, 2022). We ran post hoc pairwise comparisons, with Bonferroni‐corrected *p*‐values, using the custom function pairwise.adonis (https://github.com/pmartinezarbizu/pairwiseAdonis). We tested for homogeneity of group dispersions using the betadisper function in Vegan (Oksanen et al., 2020) and visualized the Bray–Curtis dissimilarities by making a PCoA plot using the ordinate function in Phyloseq (McMurdie & Holmes, 2013).

For testing whether (4) tissue type was a stronger predictor of microbial community composition than geographic location, we continued to analyse the β diversity of oyster tissue samples. We filtered out environmental samples (sediment and seawater samples) from our Bray–Curtis dissimilarity matrix to run a PERMANOVA of only the tissue samples, with individuals as block with 999 permutations using the adonis2 function in vegan (Oksanen et al., 2020) with two factors, geographic location (six levels: ME, MA, RI, NC, GA, and FL) and tissue type (three levels: mantle, gill, stomach). To account for the individual oyster as a level of variability, permutations were constrained to individuals using the how function in the permute R package (Simpson, 2022). We ran post hoc pairwise comparisons, tested for homogeneity of group dispersions, and visualized tissue microbiomes with PCoAs as described above, one coloured by geographic location and a second coloured by tissue type. To further assess whether tissue microbiomes had geographical patterns, we replicated the analysis we did with seawater and sediment samples, we did a correlation analysis between the first PCoA axis and latitude using the lm function in base R. We compared means of Bray–Curtis dissimilarities for comparisons between samples of the same tissue type across all geographic locations (e.g., gill from ME compared with gill from FL) and between samples of different tissue types within the same geographic location (e.g., gill form ME compared with stomach from ME) using a non‐parametric two‐sample Wilcoxon test in the ggpubr R package () and visualized these comparisons with a violin plot in ggplot2 (Wickham, 2016).

To test (5) whether each tissue type had persistent resident members of the microbiome across geographic locations, we ran a Random Forest Classification model using 10,001 trees on a feature table containing ASVs (*n* = 151) that were present in more than 10% of the samples (RandomForest R package; Liaw & Wiener, 2002). Model performance was confirmed by examining the out‐of‐bag error rate and we performed leave‐one‐out cross‐validation with 999 permutations in the caret R package (Kuhn, 2008). We visualized the results of the Random Forest model using a heatmap made with the heatmap package in R. Additionally, we performed a core analysis with a relative abundance >0.01 and prevalence >0.5, as defined in previous studies (Miller et al., 2020; Unzueta‐Martínez, Welch, & Bowen, 2022), using the core_members function, which uses abundance and prevalence thresholds to identify core ASVs, in the microbiome R package (Lahti & Shetty, 2017). We used set theory functions in R to perform list comparisons to test if any of the core ASVs were detected in the microbiomes of their surrounding environment (seawater and sediment). We visualized core ASVs with a stacked bar plot in ggplot2.

## RESULTS

**FIGURE 2:**
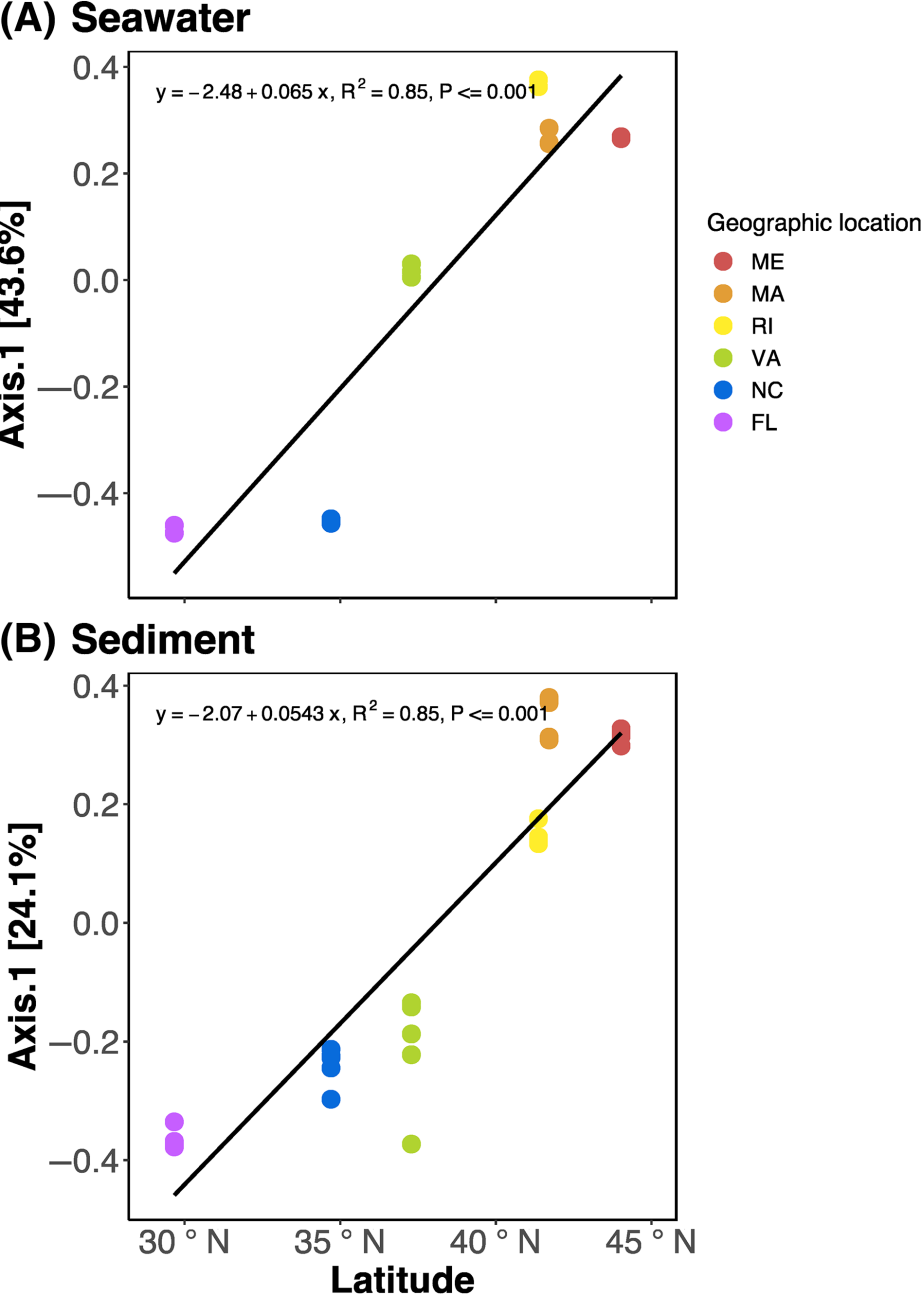
Scatter plot showing the relationship between Bray–Curtis dissimilarity distances along the first principal coordinates axis and latitude of (A) seawater and (B) sediment microbial communities in the sampled geographic locations.

We found that the β diversity of sediment and seawater microbial communities were different depending on geographic location and followed a latitudinal pattern (Figures 1B,C and 2A,B). PCoA plots of Bray–Curtis dissimilarities showed seawater (Figure 1B) and sediment (Figure 1C) microbial communities clustered according to geographic location. PERMANOVA models showed a significant effect of geographic location on microbial community composition of seawater (*p* = 1.00e−04, *R* ^2^ = 0.91) and sediment (*p* = 1.00e−04, *R* ^2^ = 0.62) communities (Table S1). In addition to finding compositionally distinct microbial communities according to geographic location, we found strong latitudinal diversity gradients in both seawater and sediment communities. The first PCoA axis of both seawater and sediment ordination plots, was significantly correlated with the latitude of our geographic sampling sites (Figure 2A,B).

**FIGURE 3:**
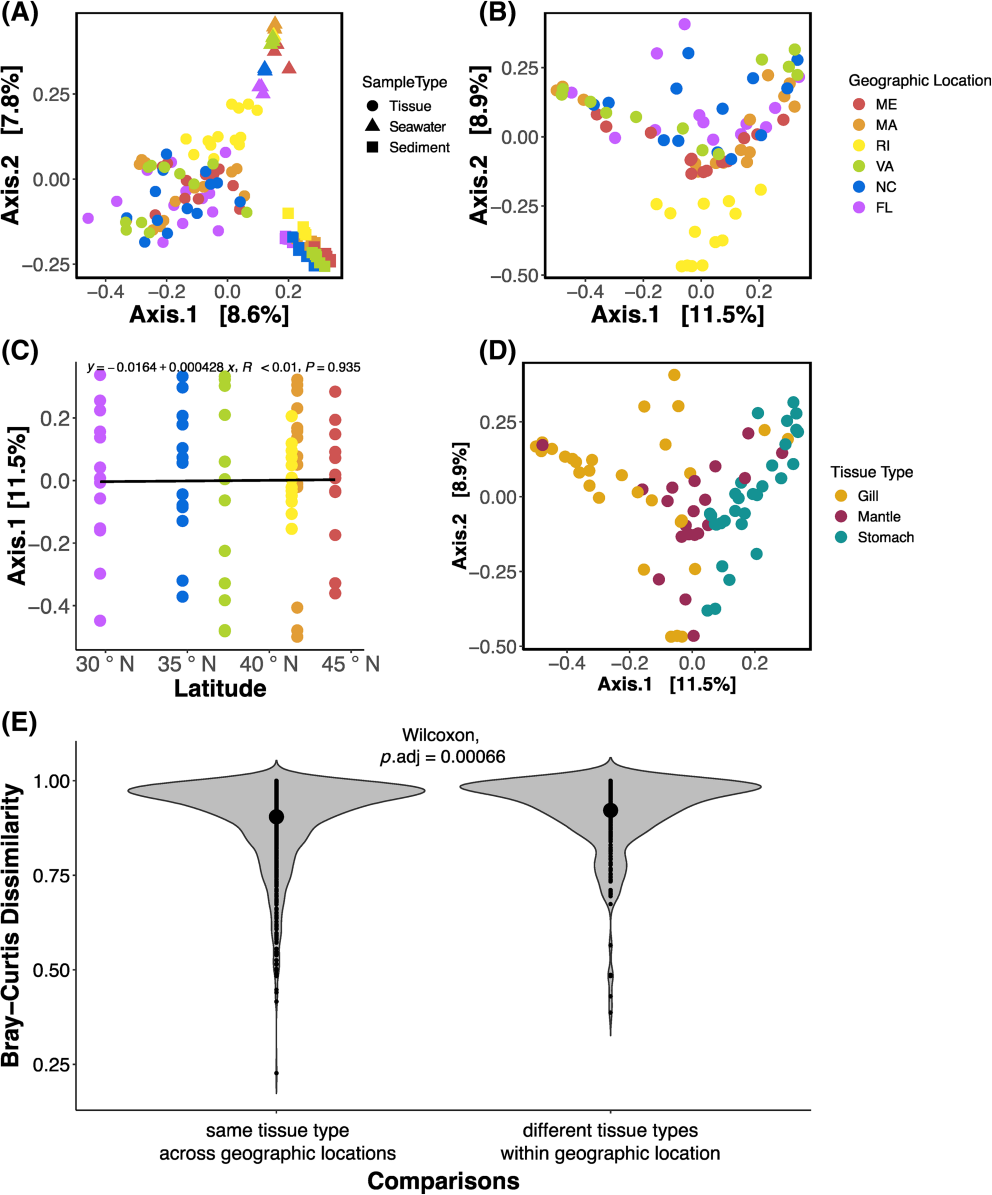
Bray–Curtis dissimilarities of microbial communities associated with oyster tissues and their surrounding seawater and sediment, visualized with principal coordinate analysis (PCoA) (A) across all sample types, and specific to oyster tissues (B) coloured by geographic location or (D) tissue type. As well as a (C) scatterplot showing no relationship between Bray–Curtis dissimilarity distances along the first principal coordinates axis and latitude of tissue‐associated microbial communities. Finally, (E) violin plot showing oyster‐associated microbial communities compared with the same tissue type across all geographic locations (e.g., gill from ME compared with gill from FL) and compared with different tissue types within the same geographic location (e.g., gill form ME compared with stomach from ME). Large dots inside the violin plot indicate group means.

Oyster‐associated microbiomes, in contrast, were different from the microbiomes in their immediate environment (seawater and sediment). A PERMANOVA model of Bray–Curtis dissimilarities, with geographic location as blocks, showed that microbial communities on oyster tissues were significantly different from those in seawater and sediments within the same geographic location (*p* = 1.00E‐04; *R* ^*2*^ = 0.14; Table S2). A PCoA plot of the same Bray–Curtis dissimilarities showed microbial communities clustered by sample type, with tight clusters for the seawater (Figure 3A, triangles) and sediment (Figure 3A, squares), whereas tissue samples (Figure 3A, circles) clustered more loosely.

A PCoA plot with colours representing geographic location showed that the microbial communities in RI oysters clustered together, whereas the other sites where interspersed (Figure 3B). We found no relationship between the first PCoA axis of the tissue ordination plot against the latitude of the sampled geographic locations (*p* = 0.94; *R* < 0.01; Figures 3C and S3). A PCoA plot with colours representing tissue type showed that the communities were organized according to tissue type (Figure 3D). We found that oyster tissue‐associated microbial communities were significantly more similar to other samples of the same tissue across geographic locations, than to different tissue types within the same geographic location (*p* = 0.001; Figure 3E; Table S3). We found a significant effect of tissue type (*p* = 0.001; *R* ^*2*^ = 0.10; Table S4), geographic location (*p* = 0.001; *R* ^*2*^ = 0.18; Table S4) and an interaction between the two factors (*p* = 0.001; *R* ^*2*^ = 0.15; Table S4). Post hoc pairwise comparisons revealed that all tissue‐type microbiomes were significantly different from each other (*p* = 0.003; Table S3), but not all geographic locations were different from each other.

**FIGURE 4:**
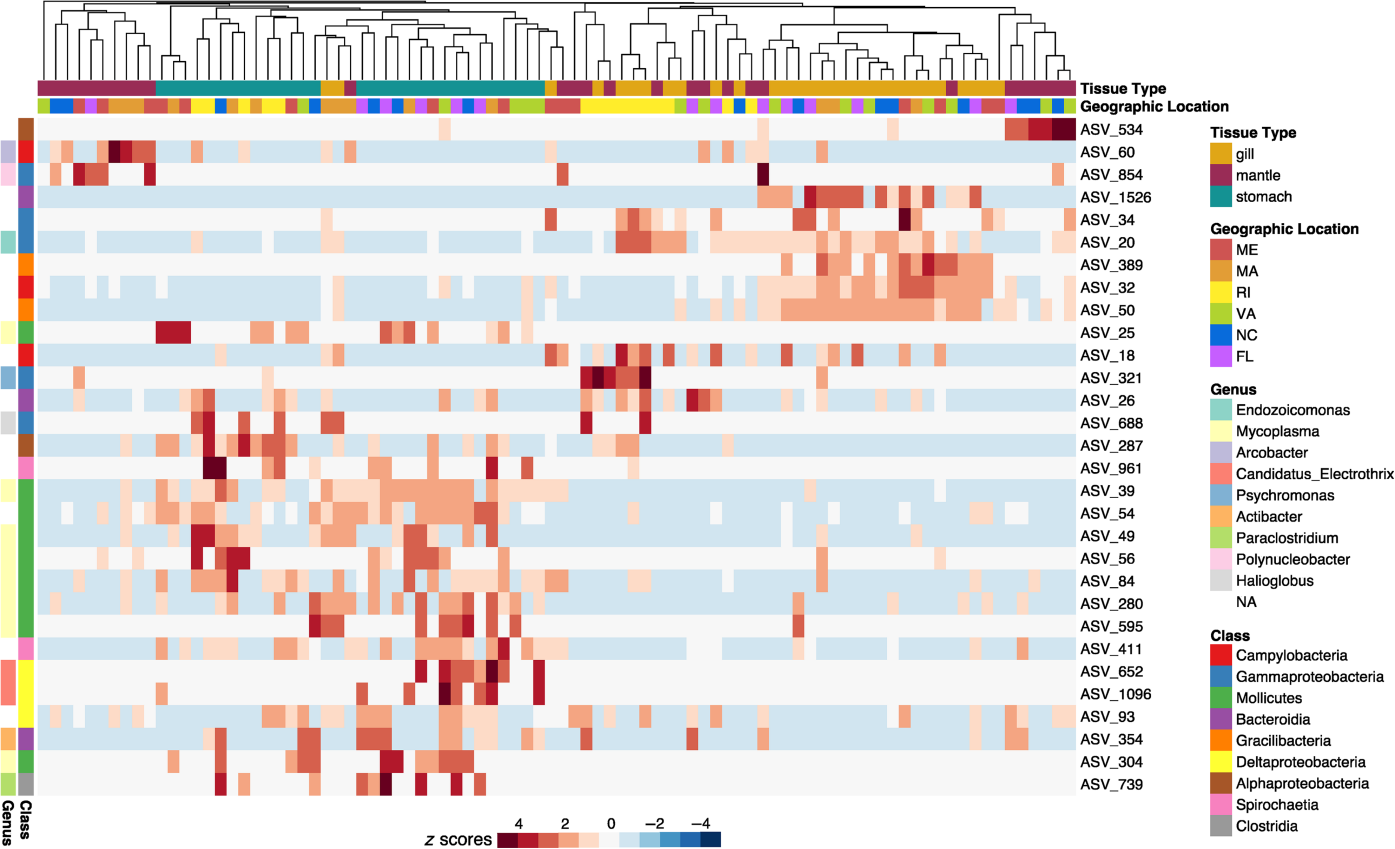
Heat map of the 30 most important amplicon sequence variants (ASVs) in contributing to a Random Forest classification model trained to predict tissue type from microbial community composition. The heat map shows the relative abundances of ASVs in samples of different tissue types, samples are clustered using Bray–Curtis dissimilarity distances. More detailed taxonomic information can be found in Table S5.

To identify resident member of the microbiome, we ran a Random Forest classification model as well as a ‘core’ microbiomes analysis. The Random Forest classification model correctly classified 72% of tissue samples as belonging to gill, mantle, and stomach with a 28% out‐of‐bag error rate. Some tissue types were predicted more easily than others based on their microbial communities, 90% of stomachs, 68.97% of mantles, and 56.67% of gills were classified correctly. We confirmed model performance with leave‐one‐out cross‐validation, with a Cohen's kappa statistic of 61.26%. The top 30 ASV that best describe each tissue type, represented nine different classes of bacteria, Mollicutes, Gammaproteobacteria, Campylobacteria, Deltaproteobacteria, Spirochaetia, Gracilibacteria, Alphaproteobacteria, Clostridia, and Bacteroidia (Figure 4).

**FIGURE 5:**
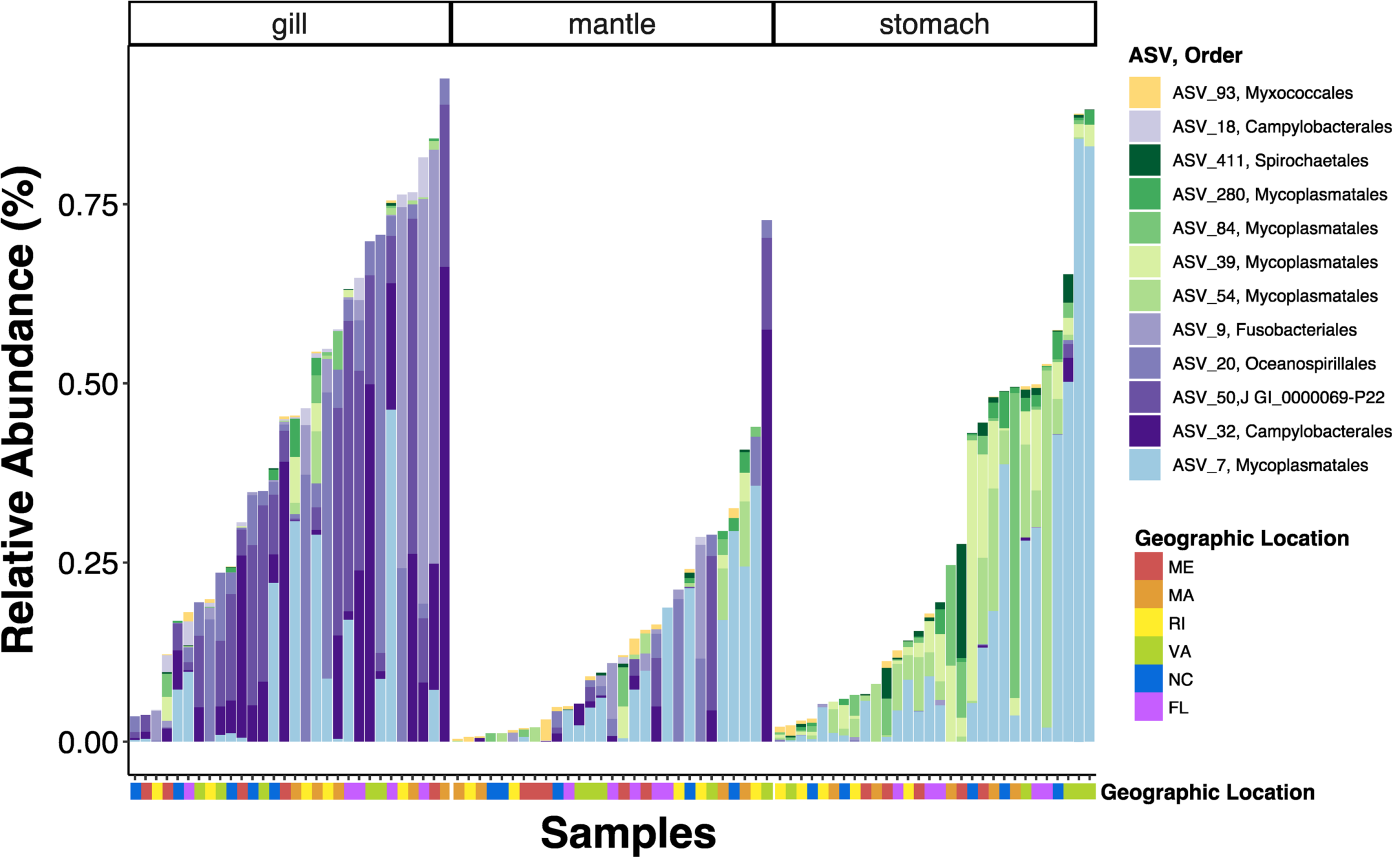
Stacked bar plot of the relative abundance of amplicon sequence variants (ASVs) identified as core members, present at >1% abundance in more than 50% of samples, in the gill (shades of purple *n* = 5), mantle (yellow *n* = 1), and stomach (shades of green *n* = 5) microbiomes. More detailed taxonomic information can be found in Table S6.

The core microbiome analysis (Figure 5) identified ASVs that were present in the same tissue type across geographic locations. We found six core ASVs in the gill microbiomes, in the orders Mycoplasmatales, Fusobacteriales, Oceanospirillales, Campylobacterales, and JGI_0000069‐P22. Among the mantle microbiomes, we identified one core ASV, ASV_93, belonging to the order Myxococcales. Among the stomach microbiomes, we identified six core ASVs, five of which belong to the order Mycoplasmatales and one to Spirochaetales. One ASV, ASV_7 in the order Mycoplasmatales, was identified as core of both the gill and stomach microbiomes. When comparing the identified core ASVs to environmental microbiomes (seawater and sediment), we found that only one ASV, ASV_9 in the order Fusobacteriales, was also detected in seawater samples, the rest of the 12 ASVs identified as core were not detected in either the seawater or sediment microbiomes. Additionally, nine out of the 12 ASVs identified as core members were also identified with the random forest model as being important taxa in defining tissue‐specific microbiomes.

## DISCUSSION

We investigated whether marine bivalves have stable, resident microorganisms inhabiting specific tissues or if their microbiomes mostly contain transient members that reflect the environmental pool of microbes. To do this, we conducted a latitudinal study of wild eastern oysters to determine whether there are resident microorganisms that persist across a wide geographical range. We characterized the microbial communities of six oyster reefs on the East Coast of the United States. We found that communities in (1) seawater and (2) sediment samples were driven by geographic location and followed latitudinal patterns of diversity, (3) oyster‐associated communities were overall distinct from seawater and sediment microbiomes, (4) microbial communities on oyster tissues of the same tissue type were more similar to each other across all geographic locations, than they were to other tissue types within the same geographic location, and (5) we identified tissue‐specific resident microbial members that persisted across geographic locations.

### Oyster‐reef seawater and sediment microbiomes differed by geographic location

Describing the biogeography of microbial communities inhabiting the surrounding environment of animals is key to understanding animal–microbiome interactions and community assembly. Free‐living microorganisms typically exhibit non‐random distribution patterns throughout space. Sediment (Green et al., 2004; Martiny et al., 2006) and seawater (Ma et al., 2022) microbial assemblages differ by geographic location and decrease in similarity with spatial separation. Our results are consistent with these geographic patterns, where site and latitude both had an effect on sediment and seawater microbial community β diversity. The geographic differentiation we observed in sediment and seawater microbiomes could be attributed to the different physical conditions in our sites along the East Coast of the United States. The oyster reefs we characterized are located within the three major current systems in the North Atlantic; the Gulf of Maine (ME and MA), the Mid‐Atlantic Bight (RI and VA), and the South‐Atlantic Bight (NC and FL), which have different geophysical characteristics like seawater temperatures (Stegmann & Yoder, 1996; Sutcliffe et al., 1976) and nutrient availability (Fennel et al., 2006) that can drive sediment (Alsterberg et al., 2011) and seawater (Morán et al., 2015) microbial community dynamics. Oyster‐reef seawater and sediment microbiomes showed distinctive biogeographic patterns that, surprisingly, didn't translate to the oyster‐associated microbiomes.

### Oyster microbiomes were different from the microbiome in their environment

Adult oyster‐associated microbial communities were distinct from the microbial assemblages in their surrounding sediment and seawater across all our sites. Previous studies reported similar findings, where oyster (Arfken et al., 2017; Diner et al., 2023) and coral (Glasl et al., 2016; Lima et al., 2023) microbiomes were distinct from their surrounding seawater and sediment microbiomes. This is particularly interesting as adult Eastern oysters play a major role in benthic‐pelagic coupling by removing suspended organic and inorganic particles from the water column and transferring them to the sediments in the form of biodeposits (Murphy et al., 2019; Newell & Jordan, 1983), or pseudofeces. Thus, oysters are intermediaries between particles suspended in seawater and the sediment, yet their gill and stomach microbiomes are highly dissimilar from the microbiomes found in reef seawater and sediments (Figure 3A; Table S2). Divergence from seawater and sediment microbial communities suggests that adult oyster tissues select for a large portion of their associated microbial communities, instead of merely reflecting the communities present in their surroundings, as seen in some invertebrates, such as caterpillars (Hammer et al., 2017). Not only were oyster microbiomes distinct from the microbiomes present in their surrounding environment, but each tissue type also had their own unique microbial composition.

### Oyster microbiomes had higher within‐tissue similarity across geographic locations than among‐tissue similarity within geographic location

Oyster microbiomes were more similar to microbiomes of the same tissue type at different geographic locations, than to other tissue types within the same geographic location. That is, a gill microbiome from Maine, for example, was more similar to a Florida gill microbiome than to either Maine mantle or stomach microbiomes. This indicates that there are tissue‐specific resident microbes that persist across a wide geographical range. Our findings are consistent with other studies that show host–species‐specific bacterial communities that persist regardless of geography (Roterman et al., 2015; Zurel et al., 2011). This pattern indicates the presence of resident members of the microbiome that may be relevant to host physiology.

### Oyster microbiomes were dictated by tissue type and geographic location

We found that oyster gill, mantle, and stomach microbiomes were significantly different depending on tissue type, despite high inter‐individual variability. It is common to find microbial community variability across tissue types (Ainsworth et al., 2015; Brodersen et al., 2018; Lokmer, Kuenzel, et al., 2016). Different tissues have distinct morphological and functional specializations which can provide unique environments for microbial colonization. Our findings are consistent with what has been previously observed in Pacific oysters (Lokmer, Kuenzel, et al., 2016) corals (Ainsworth et al., 2015), and sea grasses (Brodersen et al., 2018). In our study, this is particularly surprising because of the wide geographical range we sampled, which spans three different current systems in the North Atlantic with distinct physical and chemical characteristics, yet oyster‐associated microbial communities showed more robust differences by tissue type than by geographic location.

In addition to the strong influence of tissue type, we also observed a significant, albeit less strong effect of geographic location on oyster‐associated microbial communities. This finding supports previous studies that found relationships between geography and oyster microbiomes (King et al., 2012; Nguyen et al., 2020). It is possible that different physical characteristics of each geographic location could have influenced the oyster‐associated microbial communities, since environmental factors like temperature (Lokmer & Mathias Wegner, 2015) and pH (Scanes et al., 2021; Unzueta‐Martínez et al., 2021) also influence oyster tissue microbial communities. While oyster‐associated microbiomes were significantly different depending on geographic location, not all pairwise comparisons among locations were significant and differences by tissue type were more robust.

The interaction between tissue type and geographic location could be related to the oyster host genetics. We selected our geographic locations based on prior studies indicating substantial genetic divergence among oyster populations throughout the East Coast of the United States (Hoover & Gaffney, 2005; Hughes et al., 2017). Host genetics can influence microbial community composition and structure (Bonder et al., 2016; Goodrich et al., 2016; McKnite et al., 2012; Turnbaugh et al., 2009), so it is possible that the interaction between tissue type and geographic location were driven by the genetic differences among oyster populations. This genetic differentiation among sites may help explain why tissue samples from RI were particularly different from the rest (Figure 3B). At our RI site, there are extensive oyster restoration projects that use hatchery‐reared stocks (Jaris et al., 2019). It is possible that the wild reefs we sampled were colonized by escapees from the restoration projects, making the genetics of the RI oysters particularly different from those that came from the rest of our sampling sites. Additionally, the interaction between tissue type and geographic location could indicate the presence of transient members of the oyster microbiome. Though we did not aim to identify the transient members of the oyster microbiomes in this study, we acknowledge that they may contribute to the between‐site and between‐individual variability observed.

### Oyster tissues had resident members of the microbiome that persisted across geographic locations

We identified key microbial ASVs that differentiated oyster‐associated microbiomes by tissue type (Figure 4) and core ASVs specific to each tissue type that persisted across all geographic locations (Figure 5). Notably, the majority of identified core ASVs (11 of 12) were not detected in either the seawater or sediment microbiomes. It is possible that these ASVs were present in such low abundances in seawater and sediment samples that we did not detect them using our sampling methods. However, the microbial community composition specificity and prevalence across the large‐scale geographical range of our study indicate that these ASVs represent resident members of the oyster microbiome.

Among the gill microbiomes, four ASVs (ASV_18, ASV_20, ASV_50, ASV_32) were identified independently by both the Random Forest model (Figure 4) and core analysis (Figure 5) as key players in differentiating gill microbial communities from other tissues and having prevalent abundance patterns across all geographic locations. Two of the ASVs (ASV_18 and ASV_32) belong to the order Campylobacterales. Members of this order have been previously found associated to clams (Offret et al., 2023), urchins (Hakim et al., 2015), and Eastern oyster gills (Unzueta‐Martínez et al., 2021). Interestingly, most species of this order thrive in CO_2_ rich environments (Al‐Haideri et al., 2016; Waite et al., 2017), it is possible that CO_2_ rich microenvironments form on oyster gills as organic and inorganic particles identified as non‐edible are accumulated and embedded in mucus to be discarded as pseudofeces. Another ASV (ASV_20) belongs to the order Oceanospirillales, genus *Endozoicomonas* (Figure 5). Members of this genus are associated with a diversity of marine organisms including invertebrates like poriferans, cnidarians, molluscs, annelids, and tunicates, and vertebrates such as fish (reviewed in Neave et al., 2016). Proposed functions include nutrient acquisition and provision to their animal host (Forget & Kim Juniper, 2013; Morrow et al., 2015) and structuring of the host microbiome via secondary metabolites and probiotic mechanisms (e.g., competitive exclusion of pathogenic bacteria. Bayer et al., 2013; Jessen et al., 2013; Morrow et al., 2015). The potential nutritional and protective properties of this genus may confer benefits to the oyster host, especially when found on gill tissues. Gills are constantly in contact with the surrounding seawater and seawater‐associated microorganisms and are thought to provide an entry point for pathogens to the oyster (King et al., 2019).

Mantle microbial communities were the most variable out of all the tissues. We identified only one ASV (ASV_93 in the class Deltaproteobacteria, order Myxococcales) as being core among the mantle tissues that was also identified by the Random Forest model. Myxococcales can produce a variety of secondary metabolites and are considered one of the most important bacterial resources for the discovery of new antibiotics (Landwehr et al., 2016). Species in this order are widely distributed across environments, having been found in terrestrial sediments, freshwater lakes, marine sediments, seawater, and rarely in host‐associated environments (reviewed in Wang et al., 2021). Members of this order are micropredators that prey on other bacteria and fungi, resulting in the regulation of bacterial communities on agricultural land (Wang et al., 2020). Some can prevent and control cucumber *Fusarium* wilt by regulating the soil microbial community (Ye et al., 2020). It is possible that the Myxococcales ASVs found associated with oyster mantles in our study served as predators grazing on other microbes in the mantle tissue. To the extent that this grazing is haphazard, it could provide one explanation for the variability in the mantle tissue microbiome. There are few reports of host‐associated Myxococcales, but their ability to regulate microbial community structure through predation and production of biologically active compounds indicate that they may play a substantial role in animal microbiome composition. This variability and limited core membership of mantle tissue could be a result of the frequent exposure of the mantle to environmental microbes when feeding. Unlike gills, which use mucus to trap microorganisms and particulate matter for selective filtration (Beninger et al., 1991), mantle tissues lack this feature, leading to a broader and less selective interaction with surrounding microorganisms.

Out of six ASVs identified as core for stomach microbiomes (Figure 5), five of them (ASV_411, ASV_280, ASV_84, ASV_39, ASV_54) were also independently identified by the Random Forest model as key in differentiating stomach microbiomes from other tissue types (Figure 4). Of these, the majority (4/5) were in the class Mollicutes, order Mycoplasmatales, genus *Mycoplasma*. Members of the class Mollicutes are commonly found in association with animal guts as parasites, commensals, or symbionts (Clark, 1984; Razin, 1978; Regassa & Gasparich, 2006). Symbiotic species of the genus *Mycoplasma* play key roles in nutrition of their host by degrading recalcitrant carbon in the stomach and pancreas of marine (Wang et al., 2016) and terrestrial (Wang et al., 2004) isopods. Previous studies have found species of the genus *Mycoplasma* in high abundances in healthy oysters (Stevick et al., 2021) and abalone (Villasante et al., 2020) guts. The high prevalence and abundance of *Mycoplasma* in oyster guts across the East coast of the United States found in our study, highlights the need to investigate their functional capacity to determine if the nature of the association with oyster guts is detrimental, beneficial, or non‐consequential. One ASV (ASV_411) was identified as core for stomach microbiomes but was not identified by the Random Forest model. However, ASV_411 belongs to the Order Spirochaetales which have long been associated with the bivalve gut, in particular with the crystalline style of oysters (Husmann et al., 2010). Given this association, the identification of a Spirochete ASV in the ‘core’ group is intriguing and warrants further investigation.

## CONCLUSIONS

Our study provides compelling evidence that marine bivalves, specifically wild eastern oysters, host stable, resident microbial communities that are tissue‐specific and persist across a broad geographical range. By characterizing the microbial communities of oyster reefs along the East Coast of the United States, we found that oyster‐associated microbiomes are distinct from their surrounding seawater and sediment microbiomes. They exhibit higher within‐tissue similarity across geographic locations than among‐tissue similarity within the same location. This pattern indicates that oyster tissues select for specific resident microbes, resulting in a persistent set of resident microbiome members unique to each tissue type. These findings underscore the significant role of the oyster host in shaping its microbiome and highlight the importance of considering tissue‐specific microbial communities in future studies of bivalve‐associated microbiomes. While our results indicate distinct, tissue‐specific microbiomes that are consistent across geographic locations, the small sample size means these conclusions should be interpreted with caution. Future studies should aim to increase the number of samples to better capture the full extent of variability within and between tissue types and environmental sources. Additionally, future research should focus on exploring the functional roles of these resident microbes and their contributions to oyster health and resilience in varying environmental conditions.

## AUTHOR CONTRIBUTIONS

**Andrea Unzueta‐Martínez:** Conceptualization; methodology; investigation; validation; formal analysis; visualization; funding acquisition; writing—original draft; writing—review and editing. **Jennifer Bowen:** Conceptualization; methodology; investigation; validation; supervision; writing—review and editing; project administration.

## CONFLICT OF INTEREST STATEMENT

The authors declare no conflicts of interest.

## Supporting information


**Data S1:** Supporting information.

## Data Availability

The data that support the findings of this study are openly available in NCBI's SRA at https://www.ncbi.nlm.nih.gov/bioproject/783632?log, accession number PRJNA783632.
