# Supplementary material for: Persistent tissue‐specific resident microbiota in oysters across a broad geographical range
Source: Environ Microbiol Rep. 2024 Oct 24;16(5):e70026. doi: 10.1111/1758-2229.70026 (PMC11500617; doi:10.1111/1758-2229.70026)
Supplement: Supplementary file 1 — Data S1: Supporting information. [file EMI4-16-e70026-s001.docx]

**Table S1.** Statistical analyses of geographic location on Bray-Curtis dissimilarities of seawater and sediment microbial communities. Results of a permutational test for homogeneity of group dispersions (betadisper) of (A) seawater and (B) sediment microbial communities. Results of PERMANOVA (Adonis), using 999 permutations, for (C) seawater and (D) sediment microbial communities. The formula used for these models was: *adonis(Distance~Location, data= myadata).*

**Table S2.** Statistical analysis of microbial community Bray-Curtis dissimilarities between sample types (tissues, seawater, and sediment). (A) Results of a permutational test for homogeneity of group dispersions (betadisper) by sample type. (B) Results of PERMANOVA (Adonis), using 999 permutations, and using geographic location as blocks. The formula used for the model was: *adonis(Distance~SampleType, data= myadata, permutations = perm_control)*. (C) Results of pairwise comparisons (pairwiseAdonis).

**Table S3**.Comparison of mean Bray-Curtis dissimilarities of same tissue type across geographic locations vs. different tissue types within geographic location using a two-sample Wilcoxon test.

**Table S4**. Statistical analysis of the effects of tissue type and geographic location on Bray-Curtis dissimilarities of oyster-associated microbial communities. (A) Results of a permutational test for homogeneity of group dispersions (betadisper) by tissue type. (B) Results of a permutational test for homogeneity of group dispersions (betadisper) by geographic location. (C) Results of two-factor PERMANOVA (Adonis), using 999 permutations, and using individuals as blocks. The formula used for the model was: *adonis(Distance~Location + Tissue + Location:Tissue, data= myadata, permutations = perm_control)*. (D) Pairwise comparisons by tissue type (PairwiseAdonis). (E) Pairwise comparisons by geographic location (PairwiseAdonis).

**Table S5**. Taxonomic information, identified by comparison with the Silva database, of 30 amplicon sequence variants (ASVs) with most important contributions to a Random Forest classification model trained to predict tissue type from microbial community composition.

**Table S6**. Taxonomic information, identified by comparison with the Silva database, of ASVs identified as core members (present at greater than 1% abundance in more than 50% of samples) in the gill (*n* = 5), mantle (*n* = 1) and stomach (*n* = 5) samples.

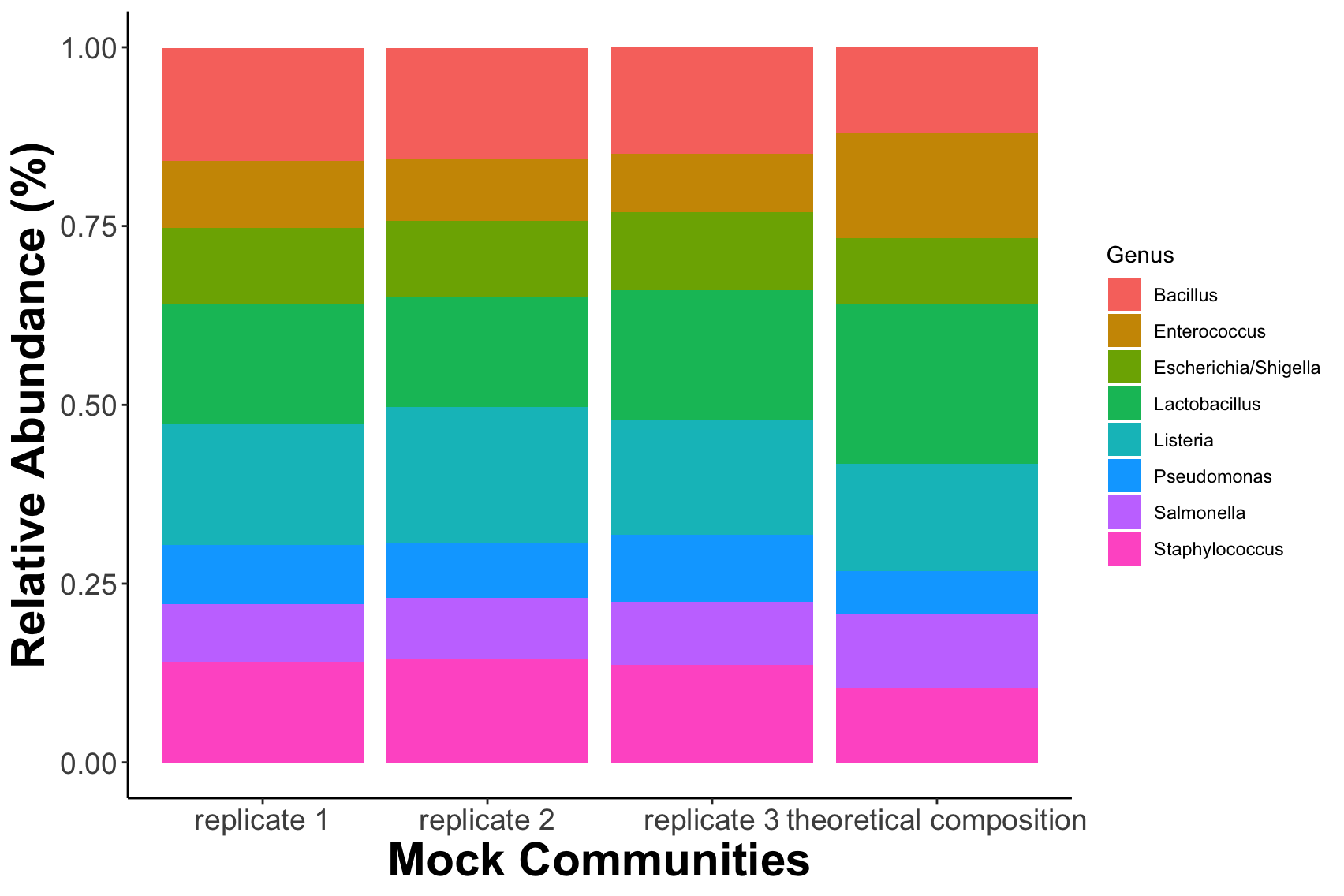


**Figure S1**. Stacked bar plot of the relative abundance of bacterial general comprising mock community replicates and the theoretical composition. Relative abundance was calculated within each sample and ASVs that made up less than 1% of the sample were excluded.


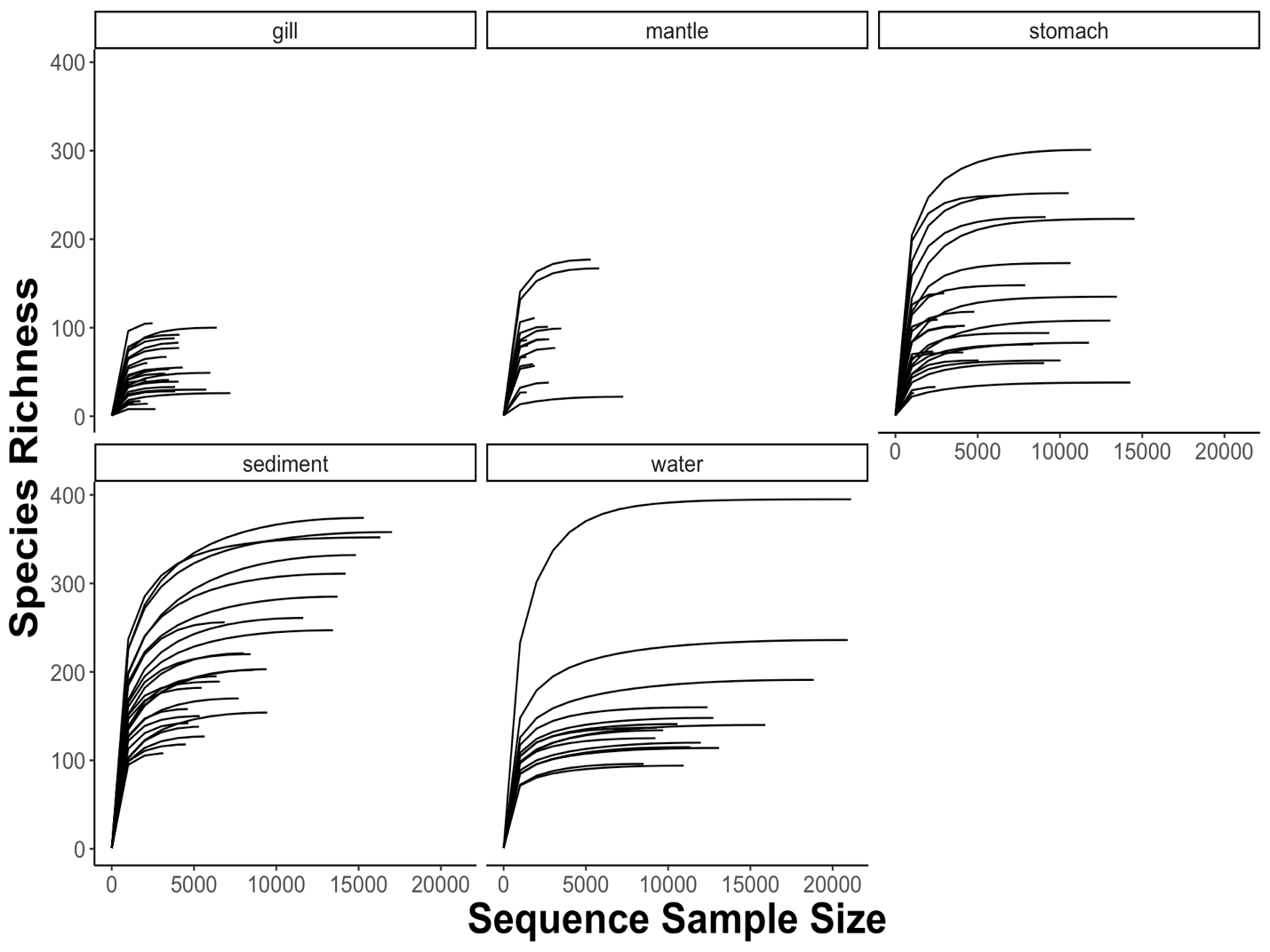


**Figure S2**. Rarefaction curves of the number of ASVs versus the number of sequences in each sample grouped by sample type.

**Figure S3.** Scatter plot showing the relationship between Bray-Curtis dissimilarity distances along the first principal coordinates axis and latitude of gill, mantle, and stomach microbial communities.
